# Impact of prior cerebrovascular disease and glucose status on incident cerebrovascular disease in Japanese

**DOI:** 10.1186/s12933-021-01367-7

**Published:** 2021-09-03

**Authors:** Momoko Oe, Kazuya Fujihara, Mayuko Harada-Yamada, Taeko Osawa, Masaru Kitazawa, Yasuhiro Matsubayashi, Takaaki Sato, Yuta Yaguchi, Midori Iwanaga, Hiroyasu Seida, Takaho Yamada, Hirohito Sone

**Affiliations:** 1 Department of Internal Medicine, Niigata University Faculty of Medicine, 1-754 Asahimachi, Niigata, Niigata 951-8510 Japan; 2 Kowa Company. Ltd, 10-4 Nihonbashi-honcho 3-chome, Chuo-ku, Tokyo, 103-0023 Japan; 3 JMDC Inc, 2-5-5 Shiba Daimon, Minato-ku, Tokyo, 105-0012 Japan

**Keywords:** Diabetes mellitus, Borderline glycemia, Epidemiology, Cerebrovascular disease

## Abstract

**Background:**

Although both a history of cerebrovascular disease (CVD) and glucose abnormality are risk factors for CVD, few large studies have examined their association with subsequent CVD in the same cohort. Thus, we compared the impact of prior CVD, glucose status, and their combinations on subsequent CVD using real-world data.

**Methods:**

This is a retrospective cohort study including 363,627 men aged 18–72 years followed for ≥ 3 years between 2008 and 2016. Participants were classified as normoglycemia, borderline glycemia, or diabetes defined by fasting plasma glucose, HbA1c, and antidiabetic drug prescription. Prior and subsequent CVD (i.e. ischemic stroke, transient ischemic attack, and non-traumatic intracerebral hemorrhage) were identified according to claims using ICD-10 codes, medical procedures, and questionnaires.

**Results:**

Participants’ mean age was 46.1 ± 9.3, and median follow up was 5.2 (4.2, 6.7) years. Cox regression analysis showed that prior CVD + conferred excess risk for CVD regardless of glucose status (normoglycemia: hazard ratio (HR), 8.77; 95% CI 6.96–11.05; borderline glycemia: HR, 7.40, 95% CI 5.97–9.17; diabetes: HR, 5.73, 95% CI 4.52–7.25). Compared with normoglycemia, borderline glycemia did not influence risk of CVD, whereas diabetes affected subsequent CVD in those with CVD- (HR, 1.50, 95% CI 1.34–1.68). In CVD-/diabetes, age, current smoking, systolic blood pressure, high-density lipoprotein cholesterol, and HbA1c were associated with risk of CVD, but only systolic blood pressure was related to CVD risk in CVD + /diabetes.

**Conclusions:**

Prior CVD had a greater impact on the risk of CVD than glucose tolerance and glycemic control. In participants with diabetes and prior CVD, systolic blood pressure was a stronger risk factor than HbA1c. Individualized treatment strategies should consider glucose tolerance status and prior CVD.

**Supplementary Information:**

The online version contains supplementary material available at 10.1186/s12933-021-01367-7.

## Background

Cerebrovascular disease (CVD) seriously affects not only mortality but also healthy life expectancy, quality of life, and economic well-being. According to a Global Burden of Diseases report, although the age-adjusted stroke mortality rates decreased globally by 36.2% between 1999 and 2016, the decline in stroke incidence remained at 8.1% [1]. Similarly, although CVD deaths are trending lower in Japan [2], stroke has many serious sequelae such as paralysis and dysarthria [3]. In addition, mean cost of post-stroke care per patient month was $1,515, which imposes a considerable economic burden [4]. Therefore, to identify patients at high risk for CVD is relevant and urgent with regard to a healthy life expectancy and the economic burden.

A history of CVD greatly increases the risk of subsequent CVD. A meta-analysis showed a recurrence rate of stroke of 3.1% at 30 days, 11.1% at 1 year, and 39.2% at 10 years after the first onset [5]. In Japan, the cumulative recurrence rates were reported to be 35.3% at 5 years and 51.3% at 10 years for stroke of all etiologies [6], and 3.81% at 1 year for ischemic stroke [7].

Diabetes as well as blood pressure is a well-established modifiable risk factor for CVD [8, 9]. A meta-analysis reported that diabetes increased the risk of ischemic stroke by approximately twofold and the risk of hemorrhagic stroke by 1.5-fold in primary prevention [10] and by 1.45-fold for secondary prevention [11]. On the other hand, there is a lack of consensus on the impact of borderline glycemia on the development of stroke [12–14].

Although diabetes mellitus was considered to be as great a risk for coronary artery disease (CAD) as a history of CAD [15], we showed that borderline glycemia had only a slight impact on CAD regardless of a history of CAD. A history of CAD increased the risk of future CAD 5–8 times whereas diabetes increased the risk of future CAD only about 2 times in Japanese men [16]. In our study, the impact of prior CAD and glucose status was evaluated in detail with a clear distinction between borderline glycemia and diabetes. However, few large studies have examined the impact of a history of CVD and glucose status on subsequent CVD development [17–19]. Therefore, we investigated the impact of glucose status and a history of CVD on subsequent CVD in Japanese men using real-world data.

## Methods

The present study retrospectively analyzed data from a nationwide claims-based database that included information on 805,992 people enrolled with a health insurance provider for company employees and their dependents in Japan. Details of the claims data and classifications were described elsewhere [20, 21]. Men aged 18–72 years who had been followed for at least 3 years between 1 April 2008 and 31 July 2016 were included and followed up to 31 August 2019. Excluded were women (n = 297,868) because of their lower incidence of CVD, individuals who were not followed at least 3 years, and those with no health examination data including blood test results (n = 144,497). Finally, data were analyzed on 363,627 men.

Participants were classified as having normoglycemia, borderline glycemia, or diabetes mellitus (DM) defined by fasting plasma glucose (FPG), HbA1c, and claims database data as follows: normoglycemia, both FPG < 5.6 mmol/L and HbA1c < 5.7% (39 mmol/mol) and no antidiabetic drug prescription; borderline glycemia, either FPG 5.6–6.9 mmol/L or HbA1c 5.7–6.4% (39–46 mmol/mol) or both and no antidiabetic drug prescription; and DM, FPG ≥ 7.0 mmol/L or HbA1c ≥ 6.5% (47 mmol/mol) or both or with an antidiabetic drug prescription regardless of FPG or HbA1c. Participants who had prior CVD at baseline and subsequent fatal or non-fatal CVD events, such as ischemic stroke, transient ischemic attack, and non-traumatic intracerebral hemorrhage, were identified according to claims using International Classification of Disease 10th revision (ICD-10) codes for CVD and medical procedures and questionnaires [22].

Categorical variables were expressed as numerals and percentages and were compared with χ^2^ tests. Continuous variables were expressed as mean ± SD or median and interquartile range. Continuous variables were compared using the unpaired Student’s t-test or Mann–Whitney U-test for two group comparisons based on their distributions. Multivariate Cox regression hazard model identified variables related to the incidence of CVD. Covariates included factors with few missing data and were not affected by blood collection time, such as age, body mass index (BMI), systolic blood pressure (SBP), low-density lipoprotein cholesterol (LDL-C), high-density lipoprotein cholesterol (HDL-C) and current smoking, in analyses of the impact of prior CVD and glucose status and their combination. We calculated the hazard ratio (HR) per 1-SD increment for several variables to directly compare the effect of traditional risk factors. Analyses were performed using SPSS (version 19.0, IBM, Chicago, IL, USA). Statistical significance was considered for P < 0.05. The Ethics Committee of Niigata University approved this study.

## Results

Baseline characteristics of our study participants according to glucose status and prior CVD are shown in Table 1. The median follow-up period was 5.2 (4.2, 6.7) years. Among 363,627 participants, 210,434, 119,933, and 33,260 had normoglycemia, borderline glycemia, and DM, respectively. Of those with normoglycemia, borderline glycemia, and DM, 1314, 1240, and 834, respectively, had prior CVD (CVD +). During the study period, 1,025, 961, and 556 CVD events occurred in CVD- and 82, 98, and 85 CVD events occurred in CVD + , respectively, in participants with normoglycemia, borderline glycemia, and DM. The incidences of CVD in CVD- and CVD + participants were 0.88 and 12.69 in those with normoglycemia, 1.46 and 16.43 in participants with borderline glycemia, and 3.11 and 21.44 in those with diabetes per 1,000 person-years, respectively.

**Table 1:** Baseline characteristics of study participants according to glucose status and prior cerebrovascular diseases

Characteristics	Normoglycemia		P-value	Border		P-value	Diabetes		P-value
	Prior CVD(-)	Prior CVD( +)		Prior CVD(-)	Prior CVD( +)		Prior CVD(-)	Prior CVD( +)	
	n = 209,120	n = 1314		n = 118,693	n = 1240		n = 32,426	n = 834	
Age (y)	43.6 ± 9.1	51.3 ± 8.8	< 0.001	48.6 ± 8.5	55.1 ± 8 .0	< 0.001	51.9 ± 8.1	56.9 ± 7.4	< 0.001
BMI (kg/m^2^)	23.0 ± 3.0	23.4 ± 3.0	< 0.001	24.2 ± 3.4	24.9 ± 3.3	< 0.001	26.2 ± 4.3	26.2 ± 4.2	0.810
SBP (mmHg)	119.8 ± 13.9	123.1 ± 14.0	< 0.001	124.1 ± 15.1	127.1 ± 15.0	< 0.001	129.9 ± 16.4	131.1 ± 15.8	0.034
DBP (mmHg)	74.8 ± 10.8	77.8 ± 10.5	< 0.001	78.4 ± 11.1	80.3 ± 10.2	< 0.001	80.9 ± 11.1	80.2 ± 10.6	0.040
HbA1c (mmol/mol)	34.1 ± 2.5	34.4 ± 2.5	< 0.001	38.4 ± 3.4	38.9 ± 3.3	< 0.001	54.3 ± 14.9	52.2 ± 13.0	< 0.001
FPG (mmol/L)	4.95 ± 0.35	5.01 ± 0.35	< 0.001	5.64 ± 0.50	5.70 ± 0.50	< 0.001	7.9 ± 2.2	7.6 ± 2.0	0.005
LDL cholesterol (mmol/L)	3.12 ± 0.78	3.02 ± 0.77	< 0.001	3.32 ± 0.80	3.11 ± 0.79	< 0.001	3.20 ± 0.84	2.92 ± 0.85	< 0.001
HDL cholesterol (mmol/L)	1.53 ± 0.38	1.52 ± 0.37	0.829	1.48 ± 0.38	1.45 ± 0.37	0.004	1.37 ± 0.36	1.38 ± 0.34	0.556
Triglycerides (mmol/L)	1.04 (0.73–1.51)	1.08 (0.78–1.55)	0.006	1.25 (0.87–1.83)	1.30 (0.93–1.83)	< 0.001	1.47 (1.02–2.19)	1.35 (0.96–2.01)	< 0.001
Current smoking (%)	78,552 (37.6)	264 (20.1)	< 0.001	44,319 (37.3)	267 (21.5)	< 0.001	13,227 (40.8)	207 (24.8)	< 0.001
History of hypertension (%)	30,960 (14.8)	574 (43.7)	< 0.001	32,190 (27.1)	734 (59.2)	< 0.001	16,057 (49.5)	632 (75.8)	< 0.001
History of dyslipidemia (%)	87,095 (41.6)	711 (54.1)	< 0.001	67,540 (56.9)	824 (66.5)	< 0.001	23,302 (71.9)	671 (80.5)	< 0.001
Medications									
β-blockers (%)	1391 (0.7)	66 (5.0)	< 0.001	2068 (1.7)	95 (7.7)	< 0.001	1576 (4.9)	127 (15.2)	< 0.001
ACEs and ARBs (%)	7750 (3.7)	353 (26.9)	< 0.001	9709 (8.2)	449 (36.2)	< 0.001	8054 (24.8)	448 (53.7)	< 0.001
CCBs (%)	7318 (3.5)	312 (23.7)	< 0.001	9715 (8.2)	421 (34.0)	< 0.001	6728 (20.7)	378 (45.3)	< 0.001
Diuretics (%)	1260 (0.6)	46 (3.5)	< 0.001	1774 (1.5)	97 (7.8)	< 0.001	1501 (4.6)	92 (11.0)	< 0.001
Statins (%)	5014 (2.4)	232 (17.7)	< 0.001	7212 (6.1)	326 (26.3)	< 0.001	7050 (21.7)	381 (45.7)	< 0.001
Antiplatelet agents (%)	879 (0.4)	327 (24.9)	< 0.001	1177 (1.0)	405 (32.7)	< 0.001	1508 (4.7)	425 (51.0)	< 0.001
OHAs (%)							13,566(41.8)	491 (58.9)	< 0.001
GLP-1 (%)							105 (0.3)	8 (1.0)	0.002
Insulin (%)							989 (3.1)	44 (5.3)	< 0.001

Data are presented as mean ± SD or median (interquartile range), n (%). International Federation of Clinical Chemistry and Laboratory Medicine units

*ACEs* angiotensin-converting-enzyme inhibitors, *ARBs* angiotensin-receptor blockers, *BMI* body mass index, *Border* borderline glycemia, *CCBs* calcium-channel blockers, *CVD* cerebrovascular disease, *DBP* diastolic blood pressure, *FPG* fasting plasma glucose, *GLP-1* glucagon-like peptide 1 receptor agonists, *HDL-C* high-density lipoprotein cholesterol, *LDL-C* low-density lipoprotein cholesterol, *OHAs* oral hypoglycemic agents, *SBP* systolic blood pressure

Hypertension was defined as SBP > 140 mmHg, DBP > 90 mmHg, or treatment for hypertension

Dyslipidemia was defined as LDL cholesterol > 3.6 mmol/L, HDL cholesterol < 1.0 mmol/L, triglycerides > 1.6 mmol/L, or treatment for dyslipidemia

As shown in Table 1, smoking rate and LDL-C were lower in the CVD + groups than in the CVD- groups. SBP, HbA1c, FPG, and triglycerides (TG) were lower in CVD- than in CVD + in those with normoglycemia and borderline glycemia, whereas HbA1c, FPG, and TG were well controlled in CVD + among DM participants. Percentages of persons with a history of hypertension and dyslipidemia and users of medication for hypertension and dyslipidemia were significantly higher in CVD + . In those with a DM status, more participants used antidiabetic agents in the CAD + than in the CAD- group.

In CVD- groups, SBP, diastolic blood pressure (DBP), HbA1c, FPG, TG, and current smoking rate tended to be high, and there was no difference in LDL-C between those with and without new CVD events during the follow-up period in each glucose category. In contrast, among CVD + , the state of control of traditional risk factors at baseline except for SBP was similar among those with or without subsequent CVD events in the borderline glycemia and DM categories (see Additional file 1: Table S1).

**Fig. 1:**
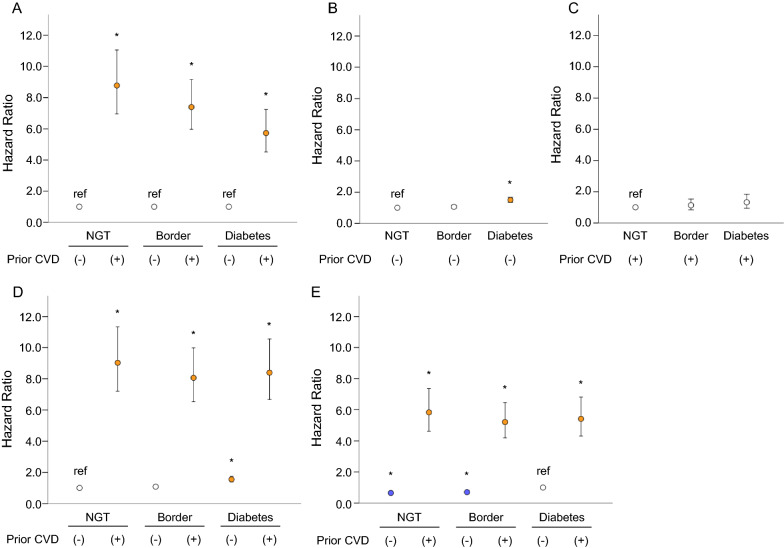
Impact of prior cerebrovascular disease and glucose status on incident cerebrovascular disease. Multivariate Cox analysis of the relationship of prior CVD and glucose status to incident CVD. **A** Impact of prior CVD on subsequent CVD according to glucose status. *P < 0.001 vs. without prior CVD. **B** Impact of glucose status on subsequent CVD in patients without prior CVD. *P < 0.001 vs. NGT. **C** Impact of glucose status on subsequent CVD in patients with prior CVD. *P < 0.001 vs. NGT. **D** Impact of prior CVD and glucose status on subsequent CVD. *P < 0.001 vs. NGT without prior CVD. **E** Impact of prior CVD and glucose status on subsequent CVD. *P < 0.001 vs. Diabetes without prior CVD. Each variable for CVD was adjusted for age, current smoking, body mass index, systolic blood pressure, LDL-C and HDL-C. Bars indicate 95% confidence intervals. Details are in Table 2. Border, borderline glycemia; CVD, cerebrovascular disease; NGT, normoglycemia

Table 2 and Fig. 1 show the multivariate-adjusted HRs for subsequent CVD events according to glucose status and prior CVD status. Compared with the CVD- groups, CVD + groups had approximately five to eightfold increases in subsequent CVD events regardless of glucose status (rows 1 and 2 from the top of Table 2 and Fig. 1A). As shown in rows 3 and 4 of Table 2 and Fig. 1B, C, the presence of borderline glycemia had no influence on future CVD events independently of prior CVD status. DM affected the incidence of CVD events only in the CVD- group. No additive effect of prior CVD and DM was observed.

**Table 2:** Relationship of prior cerebrovascular disease and glucose status to incident cerebrovascular disease

	Normoglycemia	P-value	Borderline glycemia	P-value	Diabetes	P-value
	Hazard ratio (95% CI)		Hazard ratio (95% CI)		Hazard ratio (95% CI)	
Prior CVD-	1 [Reference]		1 [Reference]		1 [Reference]	
Prior CVD +	8.77 (6.96–11.05)	< 0.001	7.40 (5.97–9.17)	< 0.001	5.73 (4.52–7.25)	< 0.001
Prior CVD-	1 [Reference]		1.06 (0.97–1.16)	0.207	1.50 (1.34–1.68)	< 0.001
Prior CVD +	1 [Reference]		1.13 (0.83–1.53)	0.433	1.32 (0.94–1.84)	0.110
Prior CVD-	1 [Reference]		1.08 (0.99–1.19)	0.088	1.55 (1.39–1.74)	< 0.001
Prior CVD +	9.03 (7.20–11.34)	< 0.001	8.06 (6.52–9.98)	< 0.001	8.39 (6.67–10.56)	< 0.001
Prior CVD-	0.65 (0.58–0.72)	< 0.001	0.70 (0.63–0.78)	< 0.001	1 [Reference]	
Prior CVD +	5.83 (4.61–7.37)	< 0.001	5.20 (4.19–6.46)	< 0.001	5.41 (4.30–6.81)	< 0.001

Each variable for CVD was adjusted for age, body mass index, systolic blood pressure, LDL cholesterol, HDL cholesterol, and current smoking

*CVD* cerebrovascular disease

**Fig. 2:**
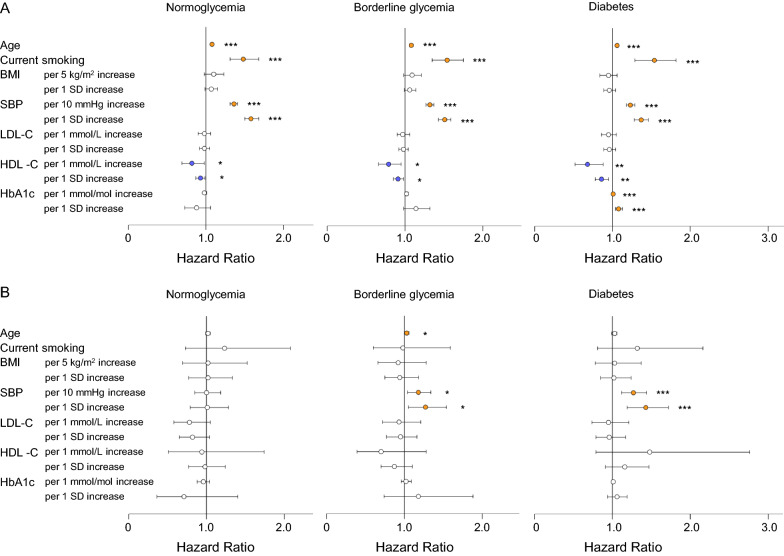
Risk factors for cerebrovascular disease according to prior cerebrovascular disease and glucose status. Multivariate cox regression analysis of traditional risk factors for the incidence of CVD according to glucose status in participants **A** without and **B** with prior CVD. Bars indicate 95% confidence intervals. *P < 0.05, **P < 0.01, ***P < 0.001. Details are in Table 3. CVD, cerebrovascular disease; SBP, systolic blood pressure

Table 3 and Fig. 2 show the HRs of each variable on for future CVD according to prior CVD and glucose status. In the CVD- groups, age, current smoking, SBP, and HDL-C were associated with the risk of incident CVD. On the other hand, in the CVD + groups, SBP was the only factor related to the risk of CVD in the presence of borderline glycemia or DM. The impact of a 1 SD increase in SBP was greater in those with normoglycemia than DM, whereas HbA1c was a significant risk factor only in the CVD-/DM group. In the CVD + /normoglycemia group, no traditional risk factor was related to subsequent CVD events. LDL-C was not a risk factor in any category according to either glycemia status or prior CVD.

**Table 3:** Impact of risk factors for cerebrovascular disease according to prior cerebrovascular disease and glucose status

	Prior CVD (-)						Prior CVD ( +)					
	Normoglycemia		Border		Diabetes		Normoglycemia		Border		Diabetes	
	HR (95% CI)	P	HR (95% CI)	P	HR (95% CI)	P	HR (95% CI)	P	HR (95% CI)	P	HR (95% CI)	P
Age (per/y increase)	1.08 (1.07–1.09)	< 0.001	1.08 (1.07–1.09)	< 0.001	1.06 (1.05–1.08)	< 0.001	1.02 (0.99–1.05)	0.122	1.03 (1.00–1.06)	0.025	1.03 (0.99–1.06)	0.125
Current smoking (yes/no)	1.48 (1.31–1.68)	< 0.001	1.54 (1.35–1.75)	< 0.001	1.54 (1.29–1.82)	< 0.001	1.23 (0.73–2.08)	0.436	0.98 (0.60–1.59)	0.927	1.32 (0.81–2.16)	0.261
BMI												
Per 5 kg/m^2^ increase	1.10 (0.98–1.23)	0.093	1.09 (0.98–1.21)	0.109	0.95 (0.84–1.06)	0.335	1.02 (0.69–1.52)	0.914	0.92 (0.66–1.28)	0.604	1.03 (0.78–1.37)	0.819
Per 1 SD increase	1.07 (0.99–1.15)		1.06 (0.99–1.14)		0.96 (0.89–1.04)		1.02 (0.77–1.33)		0.94 (0.75–1.18)		1.02 (0.85–1.24)	
Systolic blood pressure												
Per 10 mmHg increase	1.36 (1.31–1.41)	< 0.001	1.32 (1.27–1.37)	< 0.001	1.23 (1.18–1.29)	< 0.001	1.00 (0.85–1.18)	0.965	1.18 (1.04–1.34)	0.012	1.27 (1.12–1.44)	< 0.001
Per 1 SD increase	1.58 (1.50–1.68)		1.51 (1.43–1.59)		1.37 (1.28–1.46)		1.01 (0.79–1.28)		1.27 (1.05–1.54)		1.43 (1.19–1.72)	
LDL cholesterol												
Per 1 mmol/L increase	0.98 (0.90–1.06)	0.606	0.97 (0.90–1.06)	0.511	0.95 (0.86–1.05)	0.333	0.78 (0.58–1.05)	0.096	0.93 (0.72–1.21)	0.600	0.95 (0.74–1.21)	0.664
Per 1 SD increase	0.98 (0.92–1.05)		0.98 (0.92–1.04)		0.96 (0.89–1.04)		0.82 (0.65–1.04)		0.95 (0.77–1.16)		0.96 (0.79–1.17)	
HDL cholesterol												
Per 1 mmol/L increase	0.82 (0.69–0.98)	0.028	0.79 (0.66–0.95)	0.013	0.68 (0.52–0.88)	0.003	0.94 (0.51–1.74)	0.843	0.70 (0.39–1.28)	0.245	1.48 (0.79–2.76)	0.224
Per I SD increase	0.93 (0.87–0.99)		0.91 (0.85–0.98)		0.86 (0.78–0.95)		0.98 (0.77–1.24)		0.87 (0.70–1.10)		1.16 (0.91–1.47)	
HbA1c												
Per 1 mmol/mol increase	0.98 (0.96–1.01)	0.190	1.02 (1.00–1.04)	0.090	1.01 (1.01–1.02)	< 0.001	0.96 (0.88–1.04)	0.327	1.02 (0.96–1.09)	0.477	1.01 (0.99–1.02)	0.359
Per I SD increase	0.88 (0.73–1.06)		1.14 (0.98–1.32)		1.08 (1.04–1.13)		0.71 (0.36–1.40)		1.18 (0.74–1.88)		1.06 (0.94–1.19)	

Each variable for CVD adjusted for age, current smoking, BMI, systolic blood pressure, LDL cholesterol, HDL cholesterol, and HbA1c. Border, borderline glycemia; CVD, cerebrovascular disease; HR, hazard ratio; P, p-value; SD, standard deviation

## Discussion

### Main findings

The present study is the first to examine the impact of prior CVD and glucose status on subsequent CVD in a single large population. A history of CVD conferred a five to eightfold increase in subsequent CVD. Diabetes increased the risk of subsequent CVD in participants without a history of CVD whereas borderline glycemia was not related to the risk of subsequent CVD in such participants. On the other hand, neither borderline glycemia nor diabetes increased the risk of subsequent CVD in those with CVD + (Table 2). These results suggest that the impact of glucose status on CVD is small compared to a history of CVD.

### Impact of glucose status on CVD

In our previous study, the combination of CAD + and diabetes had an additive impact on the development of a new CAD events [16]. The impact of a history of CAD on subsequent CAD was stronger than that of DM, but not as strong as the impact of prior CVD on the development of a new CVD event. Huang et al. reported that elevated HbA1c was associated with an increased the risk of coronary heart disease but not with that of stroke [23]. Similarly, another meta-analysis showed that prediabetes was associated with an increased risk of coronary heart disease but not with that of stroke in patients with atherosclerotic cardiovascular disease [14]. Those findings are consistent with findings of our previous study on CAD [16] and the current study on CVD, suggesting that the impact of glucose metabolism abnormalities on CVD was modest compared with that of CAD.

### Impact of SBP on CVD

In our current study, the effect of blood pressure was much greater on CVD than that of glucose abnormality. In fact, HbA1c was a risk factor in CVD-/DM but not CVD + /DM in an analysis of traditional risk factors (Table 3). SBP was more strongly associated with CVD risk than glycemia in DM with prior CVD. These results indicate that in devising individualized treatment strategies both glucose tolerance status and prior CVD should be considered.

In those with CVD + , SBP was associated with the risk of CVD in the borderline glycemia and diabetes groups but not in the normoglycemia group (Table 3). A previous meta-analysis reported that the optimal SBP for cardiovascular disease prevention was less than 130 mmHg [24, 25]. Our results showed that the mean baseline SBP was 123.1 ± 14.0 mmHg in the normoglycemia group, 127.1 ± 15.0 mmHg in the borderline glycemia group, and 131.1 ± 15.8 mmHg in the diabetes group, which was lowest in the normoglycemia group. When baseline SBP was compared according to the presence or absence of CVD during the follow-up period, occurrence of CVD was significantly higher in those with borderline glycemia and diabetes but not normoglycemia (Supplementary Table). Thus, blood pressure in people with a history of CVD may be well controlled in the absence of abnormal glucose metabolism. The need for stringent antihypertensive treatment was shown by the United Kingdom Prospective Diabetes Study (UKPDS) 38 and the Hypertension Optimal Treatment (HOT) study [26, 27]. In the present study, although the prescription rate for antihypertensive drugs tended to be highest in DM followed by borderline glycemia and normoglycemia in those with a history of CVD, the mean SBP was higher with worsening of the glucose status. On the other hand, the rate of prescriptions for statins tended to be higher and mean LDL-C values tended to be lower with worsening of the glucose status or with a history of CVD. Histories of stroke, diabetes or their combination are important risk factors for atherosclerotic disease, and strict control of LDL-C in these patients has been recommended in guidelines [28–30]. The significance of comprehensive risk management in diabetes was demonstrated in the Steno-2 study [31, 32] and partially in J-DOIT3 [33]. The present results may reflect the situation whereby LDL-C management is generally successful but that of blood pressure remains an issue especially in patients with abnormal glucose metabolism in Japan. Recently, galectin-3 has been attracting attention as a novel therapeutic target [34]. It has the potential to influence future therapeutic strategies as well as blood pressure control.

### Impact of weight loss on CVD

Weight loss is a key factor in the prevention of cardiovascular disease. The Diabetes Prevention Program Outcomes Study showed that a lifestyle intervention involving weight loss reduced the incidence of DM [35]. UKPDS showed a modest effect of glycemic control on cardiovascular disease suppression [36] and that the suppression of cardiovascular events by metformin in overweight type 2 diabetic patients is independent of blood glucose values [37]. In the Look AHEAD trial, although weight loss did not reduce the incidence of cardiovascular disease, weight loss was associated with improvement of various risk factors for cardiovascular disease [38]. Similar results were observed in interventional trials in Japan [39, 40]. Unfortunately, our database did not include data on body weight changes. High triglyceride-glucose index levels were associated with subclinical cerebral small vessel disease in a neurologically healthy population [41] and elevated levels of adiponectin were associated with major adverse cardiovascular and cerebrovascular events and mortality risk in patients with ischemic CVD [42]. Thus, future study is needed to clarify the impact of prior CVD and glucose status on incident CVD considering these important factors.

### Strengths and limitations

The strength of our study was the combination of information on health examinations and from a claims database to assess glucose status based on clinical laboratory values and drug prescriptions and identification of CVD based on ICD-10 codes and procedures. Therefore, we were able to estimate the risk of CVD in a real-world setting on a large scale. Also of significance was that our study mainly examined the working generation, which has the greatest impact on economic activity. In addition, only cases with a long-term follow-up of at least three years were evaluated.

However, our study has some limitations. First, we excluded women because of the lower incidence of CVD. Although it was reported that the impact of impaired glucose tolerance on CVD may differ between men and women [43, 44], sex differences in incident stroke are greater in East Asia than in Western regions [45]. Thus, a larger cohort is needed to obtain a sufficient number of stroke events in East Asian women for a meaningful analysis. Second, since we do not have data from oral glucose tolerance tests (OGTTs), it is possible that the normoglycemia group included cases with impaired glucose tolerance. Previous studies showed that the impact of glucose abnormality on CVD was different between cases evaluated by fasting blood glucose and those evaluated by OGTT 2-h values [46, 47]. Although the OGTT is not always routinely performed in health check-ups and clinical settings, it is desirable to construct data that include the results of OGTTs. Third, we did not examine each subtype of CVD. Although Asians and non-Asians have the same risks of cardiovascular death, non-fatal stroke and non-fatal acute coronary syndrome, the risk of intracranial hemorrhage is 2.2 times higher in Asians [48]. In addition, diabetes has been reported to be associated with the risk of ischemic stroke and lacunar infarction, but not with other strokes [49, 50]. Therefore, a further stratified analysis is needed with regard to the subtypes of CVD. Finally, the impact of changes in glucose abnormality status, other risk factors, and treatment during follow-up could not be examined. It was reported that the transition from borderline glycemia to diabetes increases the risk of cardiovascular disease [51]. To accurately examine the risk of borderline glycemia, it would be necessary to determine whether the status of borderline glycemia persisted or progressed to diabetes during the follow-up.

## Conclusion

A history of CVD greatly increased the risk of subsequent CVD regardless of glucose status. Diabetes increased the risk of CVD in primary prevention, but less in secondary prevention. Individualized treatment strategies are needed in consideration of risk factors, such as glucose tolerance status and prior CVD.

## Supplementary Information

**Additional file 1: Table S1.** Characteristics of study participants with or without subsequent cerebrovascular diseases during the observation period according to prior cerebrovascular disease and glucose status

## Data Availability

The data that support the findings of this study are available from JMDC Inc. but restrictions apply to the availability of these data, which were used under license for the current study, and so are not publicly available. Data are however available from the authors upon reasonable request and with permission from JMDC Inc.

## Abbreviations

BMI: Body mass index
CAD: Coronary artery disease
CVD: Cerebrovascular disease
DBP: Diastolic blood pressure
DM: Diabetes mellitus
FPG: Fasting plasma glucose
HDL-C: High-density lipoprotein cholesterol
LDL-C: Low-density lipoprotein cholesterol
SBP: Systolic blood pressure
TG: Triglycerides
