# Supplementary material for: Impact of prior cerebrovascular disease and glucose status on incident cerebrovascular disease in Japanese
Source: Cardiovasc Diabetol. 2021 Sep 3;20:174. doi: 10.1186/s12933-021-01367-7 (PMC8417951; doi:10.1186/s12933-021-01367-7)
Supplement: Supplementary file 1 — Additional file 1: Table S1. Characteristics of study participants with or without subsequent cerebrovascular diseases during the observation period according to prior cerebrovascular disease and glucose status [file 12933_2021_1367_MOESM1_ESM.docx]

**Supplemental table. Characteristics of participants according to prior cerebrovascular disease, glucose status and subsequent cerebrovascular disease events**

|  | **Prior CVD-** |  |  |  |  |  |  |  |  |
| --- | --- | --- | --- | --- | --- | --- | --- | --- | --- |
|  | **Normoglycemia** |  |  | **Border** |  |  | **Diabetes** |  |  |
|  | **CVD events** |  |  | **CVD events** |  |  | **CVD events** |  |  |
|  | **(-)** | **(+)** | **p-value** | **(-)** | **(+)** | **p-value** | **(-)** | **(+)** | **p-value** |
|  | **n = 208095** | **n = 1025** |  | **n = 117732** | **n = 961** |  | **n = 31870** | **n = 556** |  |
| **Age (y)** | **43.6 ± 9.1** | **49.7 ± 8.1** | **<0.001** | **48.5 ± 8.5** | **53.0 ± 7.9** | **<0.001** | **51.9 ± 8.1** | **54.4 ± 7.5** | **<0.001** |
| **BMI (kg/m^2^)** | **23.0 ± 3.0** | **23.5 ± 3.3** | **<0.001** | **24.2 ± 3.4** | **24.6 ± 3.5** | **<0.001** | **26.2 ± 4.4** | **25.9 ± 3.9** | **0.094** |
| **SBP (mmHg)** | **119.7 ± 13.9** | **129.3 ± 17.8** | **<0.001** | **124.0 ± 15.1** | **133.0 ± 17.3** | **<0.001** | **129.8 ± 16.3** | **136.3 ± 18.8** | **<0.001** |
| **DBP (mmHg)** | **74.8 ± 10.8** | **82.7 ± 12.6** | **<0.001** | **78.4 ± 11.0** | **84.4 ± 12.2** | **<0.001** | **80.9 ± 11.0** | **83.7 ± 12.4** | **<0.001** |
| **HbA1c (mmol/mol)** | **34.1 ± 2.5** | **34.3 ± 2.6** | **0.136** | **38.4 ± 3.4** | **39.0 ± 3.6** | **<0.001** | **54.3 ± 14.8** | **56.6 ± 17.0** | **<0.001** |
| **FPG (mmol/L)** | **4.95 ± 0.35** | **4.96 ± 0.36** | **0.463** | **5.63 ± 0.50** | **5.72 ± 0.50** | **<0.001** | **7.9 ± 2.2** | **8.2 ± 2.6** | **0.001** |
| **LDL cholesterol (mmol/L)** | **3.12 ± 0.78** | **3.17 ± 0.81** | **0.042** | **3.32 ± 0.80** | **3.33 ± 0.81** | **0.691** | **3.20 ± 0.83** | **3.17 ± 0.91** | **0.437** |
| **HDL cholesterol (mmol/L)** | **1.53 ± 0.38** | **1.51 ± 0.40** | **0.193** | **1.48 ± 0.38** | **1.45 ± 0.38** | **0.011** | **1.37 ± 0.36** | **1.34 ± 0.39** | **0.040** |
| **Triglycerides (mmol/L)** | **1.04 (0.73-1.51)** | **1.15 (0.81-1.72)** | **<0.001** | **1.25 (0.87-1.83)** | **1.31 (0.94-1.94)** | **0.001** | **1.47 (1.02-2.19)** | **1.58 (1.06-2.36)** | **0.018** |
| **Current smoking (%)** | **78,089 (37.5)** | **463 (45.2)** | **<0.001** | **43,883 (37.3)** | **436 (45.4)** | **<0.001** | **12,947 (40.6)** | **280 (50.4)** | **<0.001** |
|  | **Prior CVD+** |  |  |  |  |  |  |  |  |
|  | **Normoglycemia** |  |  | **Border** |  |  | **Diabetes** |  |  |
|  | **CVD events** |  |  | **CVD events** |  |  | **CVD events** |  |  |
|  | **(-)** | **(+)** | **p-value** | **(-)** | **(+)** | **p-value** | **(-)** | **(+)** | **p-value** |
|  | **n = 1232** | **n = 82** |  | **n = 1142** | **n = 98** |  | **n = 749** | **n = 85** |  |
| **Age (y)** | **51.3 ± 8.8** | **52.1 ± 8.8** | **0.386** | **55.0 ± 8.1** | **56.7 ± 7.4** | **0.045** | **56.8 ± 7.5** | **57.6 ± 7.0** | **0.364** |
| **BMI (kg/m^2^)** | **23.4 ± 3.0** | **23.3 ± 2.6** | **0.794** | **24.9 ± 3.3** | **24.8 ± 3.4** | **0.853** | **26.2 ± 4.2** | **26.2 ± 3.8** | **0.870** |
| **SBP (mmHg)** | **123.1 ± 13.9** | **123.6 ± 15.4** | **0.739** | **126.7 ± 15.0** | **130.8 ± 14.7** | **0.009** | **130.4 ± 15.8** | **137.4 ± 15.0** | **<0.001** |
| **DBP (mmHg)** | **77.9 ± 10.5** | **77.4 ± 11.8** | **0.704** | **80.2 ± 10.2** | **81.4 ± 10.7** | **0.273** | **79.9 ± 10.5** | **82.8 ± 11.5** | **0.016** |
| **HbA1c (mmol/mol)** | **34.4 ± 2.5** | **34.1 ± 2.5** | **0.314** | **38.9 ± 3.3** | **39.2 ± 3.2** | **0.354** | **52.1 ± 12.8** | **53.9 ± 14.7** | **0.207** |
| **FPG (mmol/L)** | **5.01 ± 0.34** | **4.97 ± 0.37** | **0.262** | **5.71 ± 0.50** | **5.63 ± 0.52** | **0.157** | **7.6 ± 2.1** | **7.8 ± 1.9** | **0.432** |
| **LDL cholesterol (mmol/L)** | **3.03 ± 0.77** | **2.88 ± 0.78** | **0.077** | **3.11 ± 0.79** | **3.08 ± 0.75** | **0.709** | **2.92 ± 0.84** | **2.91 ± 0.91** | **0.964** |
| **HDL cholesterol (mmol/L)** | **1.52 ± 0.37** | **1.53 ± 0.37** | **0.883** | **1.45 ± 0.37** | **1.41 ± 0.36** | **0.361** | **1.37 ± 0.33** | **1.43 ± 0.44** | **0.148** |
| **Triglycerides (mmol/L)** | **1.07 (0.78-1.55)** | **1.11 (0.72-1.50)** | **0.533** | **1.31 (0.94-1.83)** | **1.13 (0.86-1.48)** | **0.021** | **1.35 (0.97-2.02)** | **1.32 (0.91-1.96)** | **0.586** |
| **Current smoking (%)** | **245 (19.9)** | **19 (23.2)** | **0.472** | **246 (21.5)** | **21 (21.4)** | **0.979** | **182 (24.3)** | **25 (29.4)** | **0.301** |

**Data are presented as mean ± SD or median (interquartile range), n (%). International Federation of Clinical Chemistry and Laboratory Medicine units.**

**CVD, cerebrovascular disease; DBP, diastolic blood pressure; FPG, fasting plasma glucose; Border, borderline glycemia; SBP, systolic blood pressure; SD, standard deviation**
